# Efficient and high yield isolation of myoblasts from skeletal muscle

**DOI:** 10.1016/j.scr.2018.05.017

**Published:** 2018-05-24

**Authors:** Aref Shahini, Kalyan Vydiam, Debanik Choudhury, Nika Rajabian, Thy Nguyen, Pedro Lei, Stelios T. Andreadis

**Affiliations:** a Bioengineering Laboratory, Department of Chemical and Biological Engineering, University at Buffalo, The State University of New York, Amherst, NY 14260-4200, USA; b Department of Biomedical Engineering, University at Buffalo, The State University of New York, Amherst, NY 14260-4200, USA; c Center of Excellence in Bioinformatics and Life Sciences, Buffalo, NY 14263, USA

**Keywords:** Skeletal muscle progenitors, Myoblast isolation, Satellite cells, Myogenic differentiation

## Abstract

Skeletal muscle (SkM) regeneration relies on the activity of myogenic progenitors that reside beneath the basal lamina of myofibers. Here, we describe a protocol for the isolation of the SkM progenitors from young and old mice by exploiting their outgrowth potential from SkM explants on matrigel coated dishes in the presence of high serum, chicken embryo extract and basic fibroblast growth factor. Compared to other protocols, this method yields a higher number of myoblasts (10–20 million) by enabling the outgrowth of these cells from tissue fragments. The majority of outgrowth cells (~90%) were positive for myogenic markers such as α7-integrin, MyoD, and Desmin. The myogenic cell population could be purified to 98% with one round of pre-plating on collagen coated dishes, where differential attachment of fibroblasts and other non-myogenic progenitors separates them from myoblasts. Moreover, the combination of high serum medium and matrigel coating provided a proliferation advantage to myogenic cells, which expanded rapidly (~24 h population doubling), while non-myogenic cells diminished over time, thereby eliminating the need for further purification steps such as FACS sorting. Finally, myogenic progenitors gave rise to multinucleated myotubes that exhibited sarcomeres and spontaneous beating in the culture dish.

## 1. Introduction

Satellite cells are myogenic progenitors that are located between the basal lamina and the plasma lemma of myofibers. Regeneration of adult skeletal muscle relies on the activation, proliferation and fusion of these myogenic progenitors into degenerated myofibers (Yablonka-Reuveni, 2011; Yin et al., 2013). Isolation of myogenic progenitors from the skeletal muscle niche provides us an in vitro test bed to study muscle physiology and enables studies on the intrinsic and extrinsic factors affecting myogenic differentiation (Shahini et al., 2018) as well as cellular and molecular pathways that can lead to muscle atrophy or dystrophy (Bareja and Billin, 2013). In vivo assessments of cellular function can also be done by delivering cells to the skeletal muscle of animal models in order to test their contribution to muscle regeneration after injury or disease. Such studies may lead to development of cellular therapies to promote muscle regeneration (Bareja and Billin, 2013) and combat debilitating diseases such as muscular dystrophy.

Satellite cells comprise 30–35% of the total muscle fiber nuclei at birth but decrease dramatically to 2–5% of the nuclei in adult animals which further depletes with age (Allbrook et al., 1971; Shefer et al., 2006; Day et al., 2010). Although these cells can be isolated directly from the mononuclear cell population by enzymatic digestion and titration of skeletal muscle tissue,(Rando and Blau, 1994; Hindi et al., 2017) the efficiency of this method is low due to the limited number of satellite cells released from the skeletal muscle niche (1–2 × 10^5^ myoblasts from the hindlimb muscles of one adult mouse (Yi and Rossi, 2011, Motohashi et al., 2014)). Furthermore, these protocols require passing the muscle slurry through 40 or 70 µm cell strainer, eliminating progenitor cells that may remain bonded to the myofibers (Danoviz and Yablonka-Reuveni, 2012). The majority of cells isolated using these methods are fibroblasts and other non-myogenic cell types, necessitating further purification steps by applying several rounds of pre-plating and attachment of fibroblasts to collagen coated surface (Hindi et al., 2017), fluorescence-activated cell sorting (FACS) (Yi and Rossi, 2011) or magnetic-activated cell sorting (MACS) (Motohashi et al., 2014).

Previous studies showed that satellite cells within the basal lamina of myofibers preserve their quiescent state in low serum media, while they proliferate and outgrow from their niche in high serum media (Pasut et al., 2013). The activated satellite cells (myoblasts) that migrate out of myofibers can be subcultured on the collagen coated (Calve et al., 2010; Goetsch et al., 2015) or matrigel coated dishes (Wang et al., 2014). Indeed, the combination of matrigel coating and high serum media promotes migration of myogenic progenitors from myofibers onto the tissue culture dish (Pasut et al., 2013), where they proliferate and maintain their myogenic differentiation capacity to fuse into myotubes in vitro (Shefer and Yablonka-Reuveni, 2005; Grefte et al., 2012; Pasut et al., 2013; Wang et al., 2014). This combination also provides growth advantage to myoblasts over other non-myogenic cells and maintains their differentiation capacity over prolonged culture times (Grefte et al., 2012; Wang et al., 2014).

Matrigel matrix is a protein mixture extracted from the Engelbreth-Holm-Swarm (EHS) mouse sarcoma, a mouse tumor rich in extracellular matrix proteins. Matrigel is composed of approximately 60% laminin, 30% collagen IV, and 8% entactin. Entactin interacts with laminin and collagen IV bridging these two extracellular matrix molecules and providing a structurally organized scaffold for cell attachment and proliferation. Matrigel also contains heparan sulfate proteoglycans that aid cell attachment in synergy with integrins and other adhesion receptors (Sarrazin et al., 2011), as well as growth factors such as transforming growth factor beta (TGF-β), epidermal growth factor (EGF), insulin-like growth factor 1 (IGF1), platelet derived growth factor (PDGF) and basic fibroblast growth factor (bFGF) (Hughes et al., 2010).

Here we describe a simple protocol that employs the combination of matrigel coating with culture medium containing high serum, chicken embryo extract (CEE) and bFGF to isolate myoblasts by promoting the proliferation and migration of satellite cells out of their niche. This protocol yields a high number of myoblasts (1–2 × 10^7^ myoblasts from the hindlimb muscles of one adult mouse) and eliminates downstream purification steps such as FACS or MACS. To characterize the outgrowth population, we performed flow cytometry to quantify the myogenic fraction positive for α7-integrin and non-myogenic fraction positive for SCA-1/CD31/CD45. We also performed immunostaining for the myogenic markers MyoD and Desmin and the fibroblastic marker α-smooth muscle actin (αSMA). Our results showed that ~90% of outgrowth cells were myogenic progenitors and the small fraction of non-myogenic cells could be eliminated from the culture by pre-seeding the cells on collagen coated dishes prior to seeding them on matrigel coated dishes (pre-plating). Since the combination of matrigel coating and high serum medium supplemented with bFGF and CEE provides myoblasts with growth advantage (Wang et al., 2014), the non-myogenic cells cannot overgrow in culture, yielding highly purified myogenic progenitors. In addition to proliferation, isolated myoblasts exhibit high myogenic differentiation capacity as evidenced by formation of multinucleated myotubes capable of spontaneous beating.

## 2. Materials, solutions, and methods

### 2.1. Animals

Transgenic mice with backgrounds of C57BL/6-DBA2 (Fischedick et al., 2014), C57BL/6-129Sv (Piazzolla et al., 2014), and C57BL/6-129Ola (Osorio et al., 2011) were used in this study. Mice from other backgrounds including C57BL/6 (a gift from Dr. Kenneth L. Seldeen, University at Buffalo, NY), and FOXn1nu nude (a gift from Dr. Natesh Prashurama, Universtity at Buffalo, NY) were also used to demonstrate broader applicability of this protocol. All animals were maintained in University at Buffalo laboratory animal facility according to the guidelines of Institutional Animal Care and Use Committee (IACUC) at the University at Buffalo. Animals at different ages (4 weeks to 2 years old) were euthanized in standard CO2 chambers and hindlimb muscles were dissected up to 4 h post sacrifice.

### 2.2. Matrigel coating

Cell culture dishes or flasks were cooled down to 4 °C and matrigel coating was performed on ice. Matrigel at 8–9 mg/ml (depending on the lot number, CORNING, Corning, NY) was diluted in phosphate buffer saline (PBS) to a working concentration of 0.9 mg/ml or 0.09 mg/ml (Note 4). Diluted matrigel solutions were added to the plate at 0.05 ml/cm^2^ corresponding to 45 µg/cm^2^ or 4.5 µg/cm^2^, respectively and kept at 4 °C for 10 min. Subsequently, the solution was removed and the flasks were incubated at 37 °C for 1 h before seeding cells.

### 2.3. Collagen coating

To coat the tissue culture dishes or flasks with collagen, the dishes were incubated overnight at 4 °C with 0.1 mg/ml solution of type I rat tail collagen (CORNING) in sterile water at 0.05 ml/cm^2^ or 5 µg/cm^2^. The solution was aspirated and the plates were allowed to dry before seeding cells.

### 2.4. Stock solutions for enzymatic digestion

Stock solutions for enzymes were reconstituted in sterile PBS to the following concentrations: 5000 U/ml collagenase type II (Sigma-Aldrich, St. Louis, MO), 150 U/ml collagenase D (Sigma-Aldrich), 250 U/ml dispase II (Sigma-Aldrich). A stock of 250 mM CaCl_2_ in water was also prepared. The stock solutions were stored in −20 °C. The final enzymatic solution mix was PBS containing collagenase type II (500 U/ml), collagenase D (1.5 U/ml), dispase II (2.5 U/ml), and CaCl_2_ (2.5 mM).

### 2.5. Cell culture medium

The medium that was employed for the isolation and proliferation of myogenic progenitors (proliferation medium, PM) was composed of high glucose Dulbecco's Modified Eagle Medium (DMEM, Gibco, Grand Island, NY), 20% fetal bovine serum (FBS, Atlanta Biologicals, Flowery Branch, GA), 10% horse serum (HS, Gibco), 0.5% chicken embryo extract (CEE, Accurate Chemical and Scientific, Westbury, NY), 2.5 ng/ml bFGF (ORF Genetics, Iceland), 10 µg/ml gentamycin (Gibco), and 1% Antibiotic-Antimitotic (AA, Gibco), and 2.5 µg/ml plasmocin prophylactic (Invivogen, San Diego, CA). Differentiation medium (DM) containing DMEM with high glucose, 5% HS and 1% AA was used to promote formation of multinucleated myotubes.

### 2.6. Isolation protocol

**Fig. 1:**
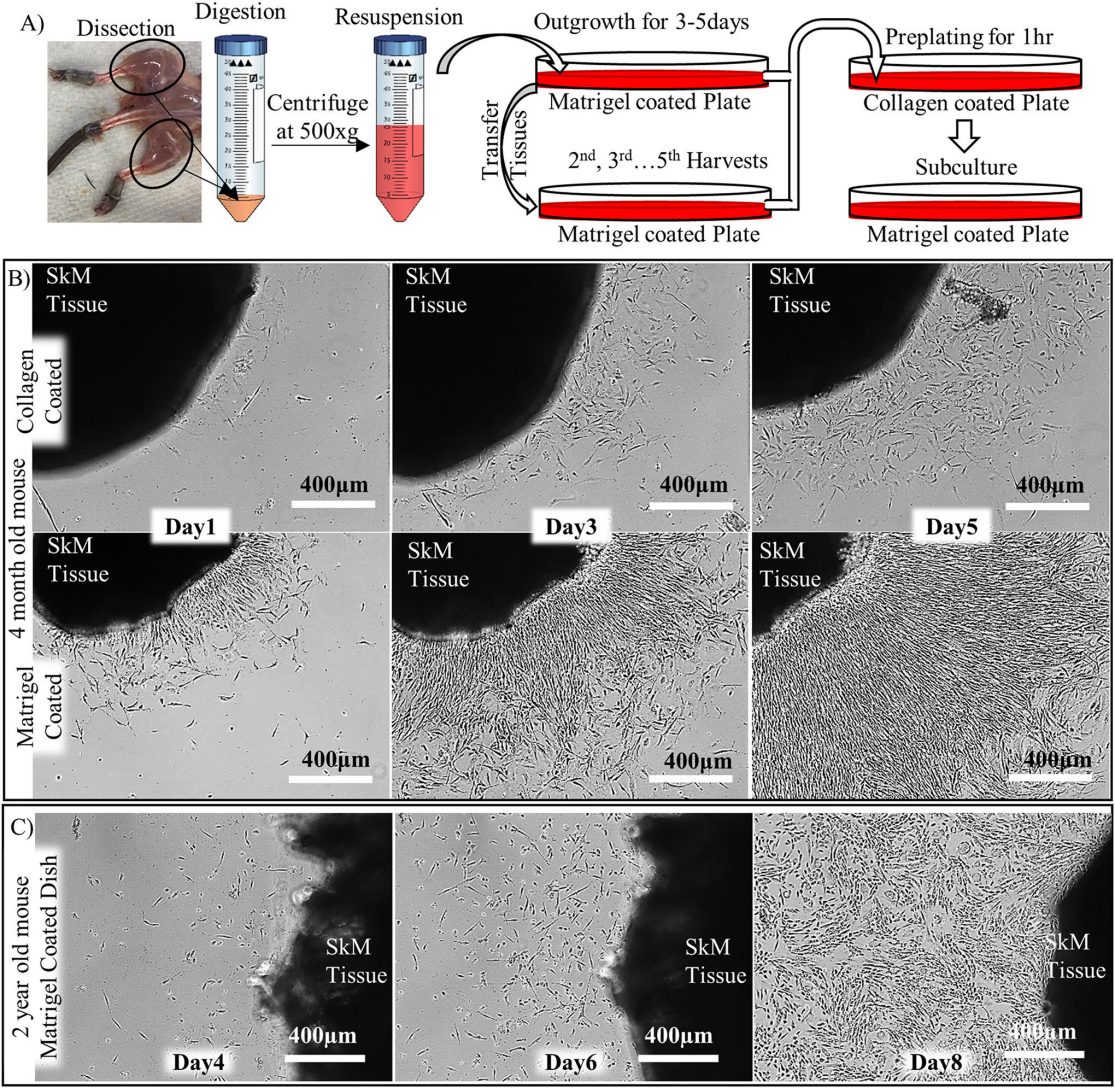
Outgrowth of cells from skeletal muscle tissue explants. (A) Schematic representation of the protocol for myoblast isolation. (B) Outgrowth of cells from the skeletal muscle tissue of a 4-month old mouse on matrigel-coated vs. collagen-coated dishes at 1, 3, and 5 days after seeding. (C) Outgrowth of cells from the skeletal muscle tissue of a 2-year old mouse on matrigel-coated dish.

The video and schematic of myoblast isolation process are shown in Supplementary Video 1 and Fig. 1A, respectively, and described below:

1. Sacrifice the mice using CO_2_ asphyxiation.
2. Spray the mice with 70% ethanol and transfer the mice to a sterile hood.
3. Cut the skin in the back region and peel it to completely expose the hindlimb muscles.
4. Isolate skeletal muscle from the hindlimbs and carefully discard pieces of fat.
5. Mince the muscle tissues to small pieces and transfer them to a 50 ml conical tube.
6. Digest the small pieces of tissue in 1 ml enzymatic solution and incubate for 60 min in 37 °C water bath while agitating the tube every 5 min (Note#1).
7. Centrifuge at 300 × *g* for 5 min and resuspend the pellet in proliferation medium (PM).
8. Seed the suspension containing small pieces of muscle tissue on matrigel coated flasks at 10–20% surface coverage and incubate at 37 °C and 10% CO_2_ to allow attachment of the tissues to the surface and subsequent migration of cells.
9. Observe the cells every two days by gently moving the flasks under phase or brightfield microscope. If there is no local confluence, add ~0.1 ml/cm^2^ medium to the flasks and return them back in the incubator. When local confluence is observed (Note#2), transfer the tissues to a new flask according to step#10 and pass the cells according to step#11.
10. To transfer the tissues to a new flask, first shake the flask to detach the tissues from the surface, and then transfer the medium containing suspended pieces of tissue to 50 ml conical tubes. Wash the surface with PBS and add the wash solution to the same conical tubes. Centrifuge at 300 × *g* for 5 min, resuspend the tissues in fresh PM, and seed the tissues on a matrigel coated flask to allow for another round of myoblast outgrowth. This process can be repeated several times; however, the population of myogenic progenitors decreases after the 3rd–4th round of harvest.
11. To pass the isolated cells, detach the cells using 0.25% trypsin and centrifuge at 300 × *g* for 5 min. The isolated cells can be frozen in PM supplemented with 10% DMSO or can be seeded at 3000 cell/cm^2^ for further expansion (Note#3). Perform pre-plating in the first few passages according to step#12 to eliminate the epithelial or fibroblastic cells from the myoblast culture.
12. For pre-plating, seed cells on collagen-coated flasks at the density of 10,000 cell/cm^2^ and incubate at 37 °C and 10% CO_2_ for 1 h. Then transfer the supernatant to a matrigel-coated dish (Note#4).
13. To induce differentiation into multinucleated myotubes, plate cells at 10,000 cell/cm^2^ on plastic coverslip chambers that are coated with matrigel (Note#5). Although upon confluence the cells spontaneously form multinucleated tubes in PM, switching to DM enhances myotube formation significantly.

**Note 1** Enzymatic digestion is not necessary for isolation, as the minced tissues without enzymatic digestion also released myoblasts after 1 week. However, enzymatic digestion expedites the process of cellular outgrowth significantly (Fig. S1A–B).

**Note 2** Do not keep the cells in local confluence as this increases the chance of spontaneous differentiation of myoblasts into myotubes (Fig. S1B).

**Note 3** Presence of bFGF is essential to maintaining the undifferentiated state of myoblasts and successful expansion of these cells (Fig. S2A). CEE also enhanced the outgrowth and proliferation of myoblasts and is known to play important role in myoblast culture (Gharaibeh et al., 2008).

**Note 4** Coating tissue culture dishes with higher concentration of matrigel (0.9 mg/ml; 45 µg/cm^2^) resulted in higher myoblast outgrowth as compared to lower concentration (0.09 mg/ml; 4.5 µg/cm^2^) (Fig. S1A). On the other hand, low matrigel concentration worked equally well when cells were subcultured. Matrigel is necessary for culturing myoblasts as the cells seeded on collagen do not spread and migrate well on the surface (Fig. S2B).

**Note 5** Despite the higher resolution of imaging on glass, the myogenic progenitors did not perform well on the glass chambers, which may be due to lower binding of matrigel proteins to glass as compared to plastic.

## 3. Results

### 3.1. Outgrowth of myoblasts from skeletal muscle tissues

**Fig. 2:**
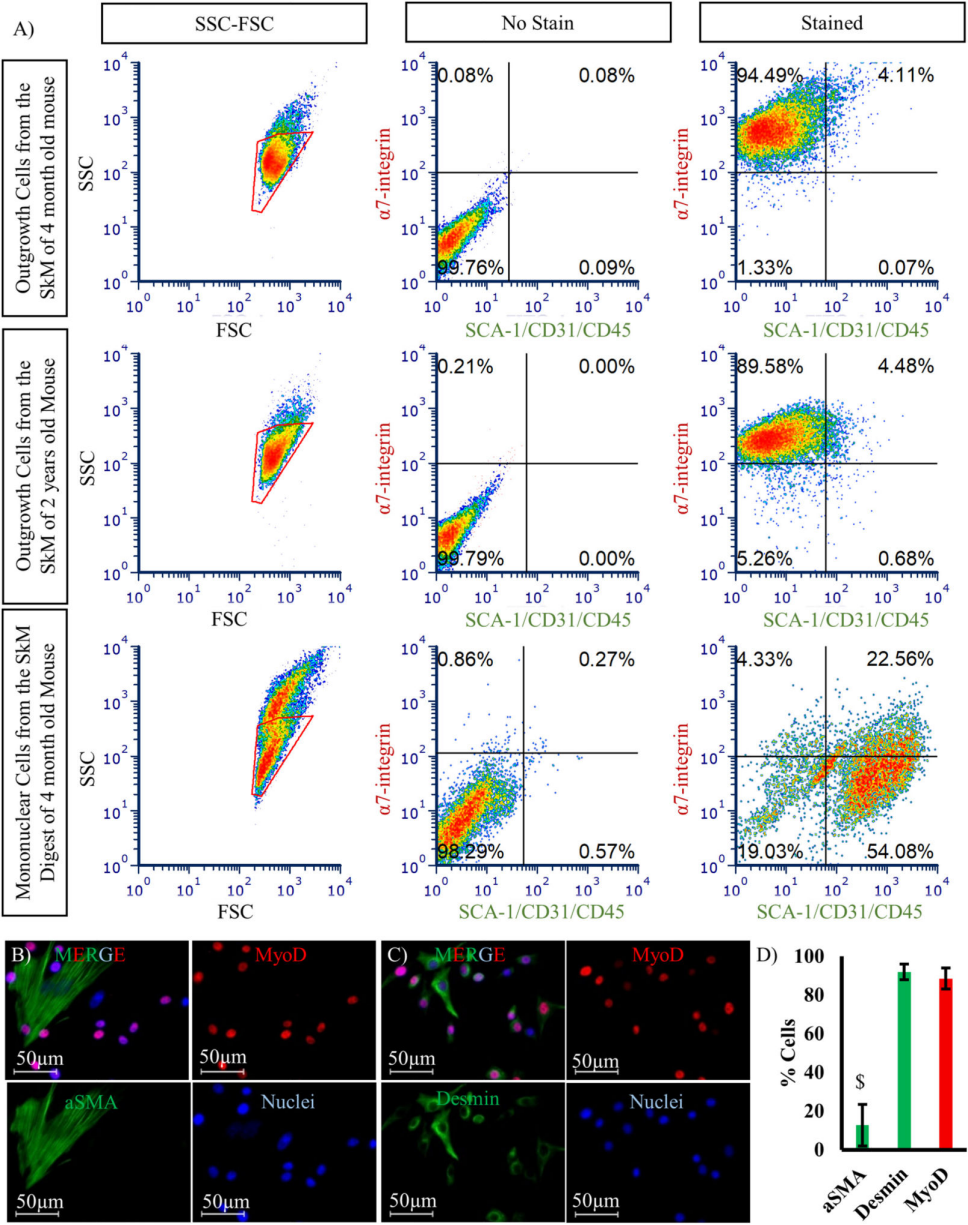
Characterization of outgrowth cells from skeletal muscle tissue on matrigel-coated dish. (A) Flow cytometry analysis of outgrowth cells from a 4-month and a 2-year old mouse as well as mononuclear cells from the skeletal muscle digest of the 4-month old mouse stained with APC conjugated α7-integrin antibody and FITC conjugated SCA-1, CD31, and CD45 antibodies. Immunostaining of outgrowth cells from a 4-month old mouse for (B) αSMA (green) and MyoD (red); (C) Desmin (green) and MyoD (red); cell nuclei were counterstained with Hoechst 33342. Scale bar = 50 µm. (D) Quantification of the percentage of cells stained for αSMA (12 ± 10%), Desmin (92 ± 4%) or MyoD (88 ± 5%). Data are shown as mean ± standard deviation (n = 80 cells from 7 fields of view; $ denotes p < 0.05 as compared to others). (For interpretation of the references to colour in this figure legend, the reader is referred to the web version of this article.)

Seeding the skeletal muscle slurry on matrigel-coated dishes in the presence of PM (Fig. 1A) enabled migration of myoblasts out of the tissues and subsequent proliferation (Fig. 1B). Migration was enhanced with increased surface concentration of matrigel from 4.5 µg/cm^2^ to 45 µg/cm^2^ (Fig. S1A). Interestingly, donor aging resulted in delayed cellular outgrowth as cells could be seen as soon as one day post-seeding of tissues from 4-month old mice as compared to 4–6 days for 2-yr old mice (Fig. 1B–C). The outgrowth cells contained mostly small compact cells, most likely myoblasts, and few large and flat cells that were most likely fibroblasts. Flow cytometry analysis showed that ~90% of the outgrowth cells were myogenic progenitors positive for α7-integrin and negative for SCA-1/CD31/CD45, while only 1–5% of the total mononuclear cells from the skeletal muscle digest were myogenic progenitors (Fig. 2A). Furthermore, immunostaining for myogenic markers (MyoD, and Desmin) and fibroblastic marker αSMA showed that the vast majority of isolated cells were positive for myogenic markers. Specifically, 88 ± 5% were positive for MyoD, 92 ± 4% for Desmin, and 12 ± 10% for αSMA (n=80 cells from 7 fields of view; $ denotes p < 0.05 as compared to others) (Fig. 2B–D).

After outgrowth, the cells must be replated to a lower density (3000 cell/cm^2^) to avoid local confluence, which results in differentiation of myoblasts into multinucleated myotubes (Fig. S1B). Besides matrigel coating, bFGF was also essential to maintaining the undifferentiated and proliferative phenotype of myoblasts, as bFGF removal resulted in significantly increased spontaneous differentiation (Fig. S2A–B).

In contrast to matrigel, minimum outgrowth of cells was observed on collagen-coated dishes (Fig. 1B). In addition, only 13% of the outgrowth cells on collagen were myogenic progenitors positive for α7-integrin and negative for SCA-1/CD31/CD45 (Fig. S3A) but most cells stained positive for fibroblastic marker αSMA (57 ± 9%) and only a small portion was positive for the myoblast markers MyoD (6 ± 4%) and Desmin (10 ± 4%) (n = 30 cells from 7 fields of view; $ denotes p < 0.05 as compared to others) (Fig. S3B–D).

### 3.2. Purification of skeletal muscle progenitors

**Fig. 3:**
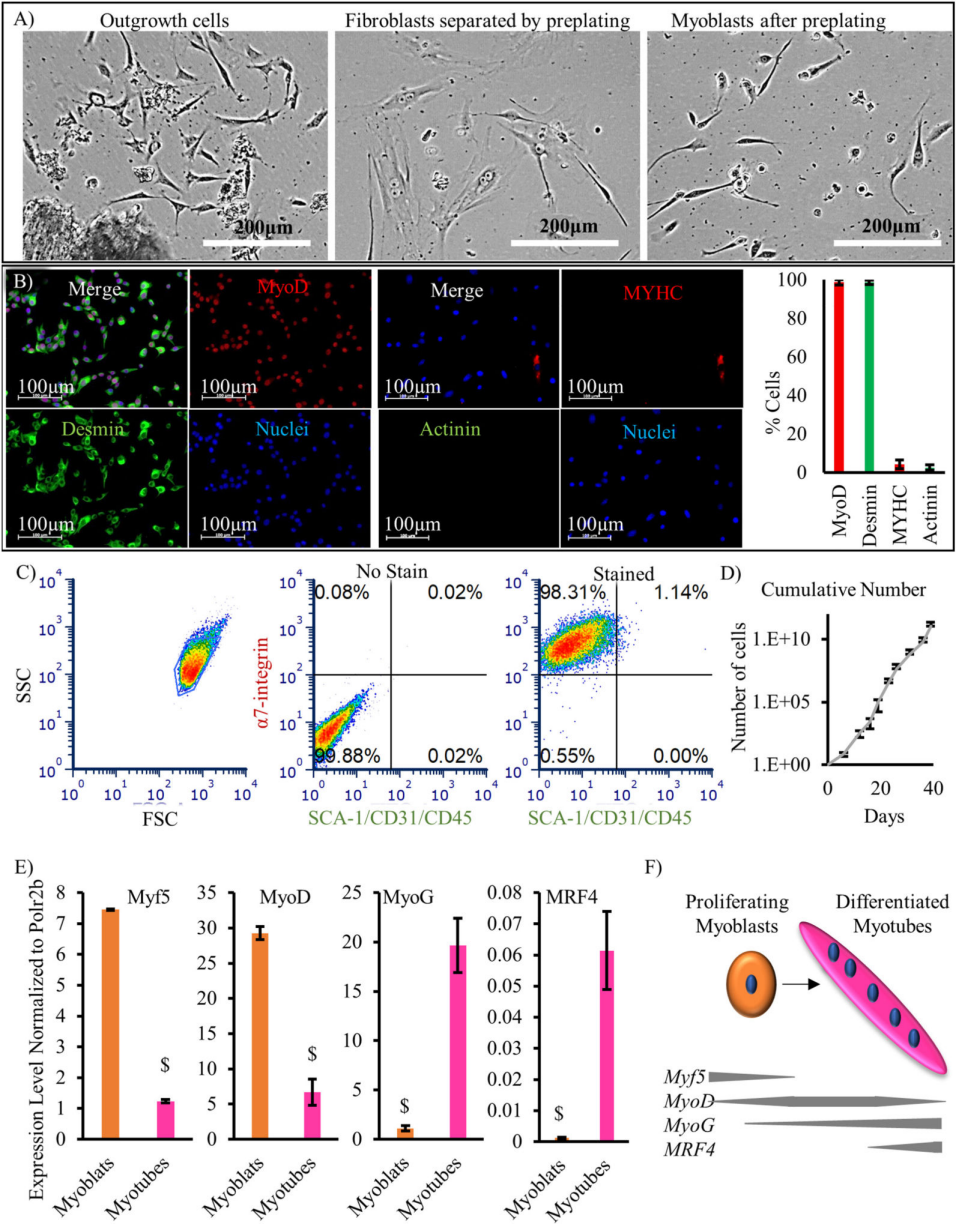
Characterization of myoblasts after outgrowth and pre-plating. (A) Representative phase contrast images showing the morphology of skeletal muscle outgrowth cells (left), fibroblasts adhered onto collagen after short-term incubation (pre-plating, middle), and purified myoblasts after pre-plating (right). (B) Immunostaining for MyoD, Desmin, MYHC, and Actinin (scale bar = 100 µm) show that 98.7 ± 1.1% of cells are positive for MyoD, 98.7 ± 1.3% are positive for Desmin, 4 ± 2% are positive for MYHC, and 3 ± 1% are positive for Actinin (n = 700 cells from 7 fields of view). (C) Flow cytometry analysis with APC conjugated α7-integrin antibody and FITC conjugated SCA-1, CD31, and CD45 antibodies shows that ~98% of the cells are myogenic progenitors (APC+/FITC−). (D) Growth curve of myoblasts. The results are shown in a log-linear plot of the cumulative cell number as a function of time; the slope of the curve was used to calculate the doubling time (24 ± 1 h). Data are shown as mean ± standard deviation (n = 3 independent experiments). (E) Expression of *Myf5, MyoD, MyoG*, and *MRF4* genes normalized to the house keeping gene *Polr2b* in myoblasts prior to differentiation and myotubes after differentiation ($ denotes p < 0.05 as compared to all other samples). (F) Schematic showing expression of transcription factors at different stages of myogenic differentiation from quiescent satellite cells to multinucleated myofibers.

While PM provides proliferation advantage to myoblasts over non-myogenic cells, we attempted to further separate the non-myogenic cells from myoblasts, by exploiting the differential attachment of fibroblasts to collagen. Specifically, when plating the cells on collagen-coated dishes for 1 h, fibroblasts bound to collagen faster, while myoblasts remained in the supernatant and could be transferred to matrigel-coated dishes for further expansion (Fig. 3A). Indeed, after only one round of pre-plating, 98 ± 1% of the cells were positive for myoblast markers MyoD and Desmin, while a small population of 4 ± 2% were positive for myotube markers MYHC and Actinin (n = 700 cells from 7 fields of view) (Fig. 3B). Flow cytometry also showed that ~98% of the cells were positive for α7-integrin and negative for SCA-1/CD31/CD45 (Fig. 3C). The purified myoblasts adhered well to matrigel coated dishes (Fig. S2B) and proliferated with an average doubling time of 24 ± 1 h for up to 40 days in culture (~10 passages) (Fig. 3D). qRT-PCR for myogenic regulatory factors confirmed that the early markers decreased with differentiation of myoblasts into myotubes (*Myf5*: Myoblasts 7.44 ± 0.02 vs. Myotubes 1.23 ± 0.05 and *MyoD*: Myoblasts 29.25 ± 0.91 vs Myotubes 6.68 ± 1.86), while late markers increased (*MyoG*: Myoblasts 1.10 ± 0.27 vs. Myotubes 19.67 ± 2.74 and *MRF4*: Myoblasts 0.0012 ± 0.0002 vs. Myotubes 0.061 ± 0.012) (Fig. 3E&F).

### 3.3. Differentiation

**Fig. 4:**
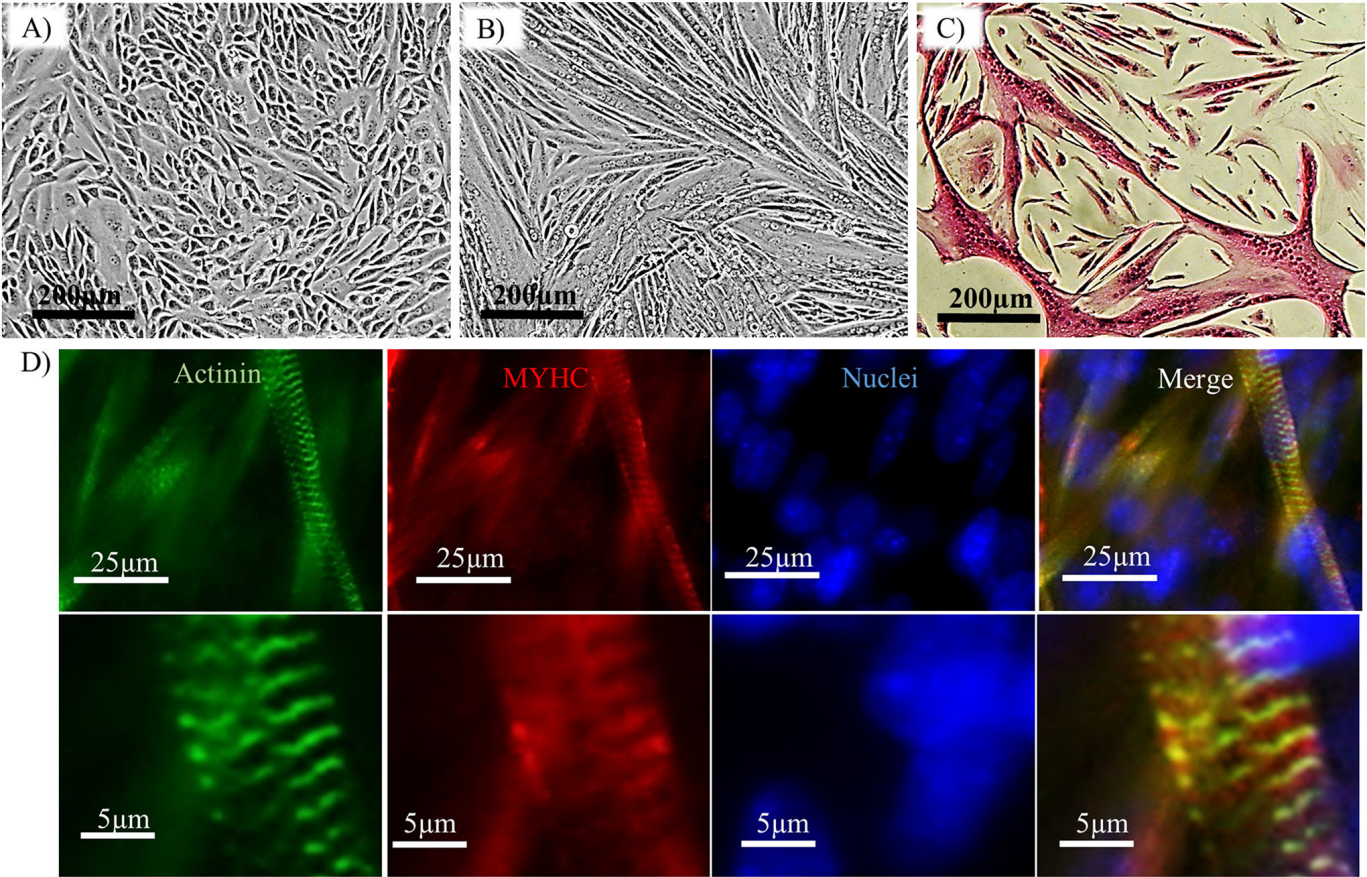
Differentiation of myoblasts into multinucleated myotubes. (A) Morphology of myoblasts after reaching 100% confluence; scale bar = 200 µm. (B) Myotubes formed after 5 days of induction in differentiation medium (DM); scale bar = 200 µm. (C) Staining of myotubes for Hematoxylin and Eosin (H&E). (D) Immunostaining of myotubes for actinin (green) and myosin heavy chain (MYHC, red) confirms the presence of sarcomeres on the 5th day of differentiation. (For interpretation of the references to colour in this figure legend, the reader is referred to the web version of this article.)

After cells reached confluence (Fig. 4A), they were coaxed to differentiate by treatment with DM for 5 days. Myotubes could be easily observed by phase contrast microscopy (Fig. 4B) and H&E staining (Fig. 4C) as well as immunostaining for myosin heavy chain (MYHC) and actinin, two muscle specific contractile proteins (Fig. 4D). Spontaneous beating was also observed, indicating development of contractile function (Supplementary Video 2).

## 4. Discussion

Satellite cells are myogenic progenitors that comprise a small portion of mononuclear cells in skeletal muscle tissue (Icronimakis et al., 2007). To study the physiology of skeletal muscle in vitro, these cells can be isolated, expanded and differentiated to multinucleated myofibers. Herein we described a detailed protocol for efficient isolation of myogenic progenitors that were characterized by expression of α7-integrin and myoblastic markers, MyoD and Desmin, as well as their ability to form multinucleated and beating myotubes. Notably, we applied the protocol to isolate myoblasts from mice from various backgrounds such as C57BL/6-DBA2, C57BL/6-129Sv, C57BL/6-129Ola, C57BL/6, and FOXn1nu nude mice and observed a comparable yield among all of them (data not shown).

Direct isolation of myogenic progenitors from skeletal muscle slurry results in a low yield of 1–2 × 10^5^ cells from the hindlimb muscles of one mouse (Yi and Rossi, 2011; Motohashi et al., 2014) (Fig. 2A), possibly due to the small size of the skeletal muscle satellite cell pool (Icronimakis et al., 2007) and their protected location underneath the basal lamina of myofibers. Therefore, many myogenic progenitors attached to myofibers may get filtered out by cell strainer. In contrast, our protocol does not solely rely on the small population of myogenic progenitors that are dissociated with enzymatic digestion but takes advantage of the outgrowth of myogenic progenitors from skeletal muscle tissue explants onto matrigel resulting in much higher yield of 1–2 × 10^7^ myoblasts from the hindlimb muscles of one mouse (Fig. 1 and Supplementary Video 1). Although enzymatic digestion was not necessary for isolation of cells from minced tissues, it accelerated cellular outgrowth, possibly due to the higher surface area of digested myofibers in contact with the matrigel-coated surface (Fig. S1).

We observed that donor aging delayed migration of myoblasts out of their niche (Fig. 1C) and decreased the total number of outgrowth cells. This observation is in line with the previous studies reporting that the aging muscle contains fewer myogenic progenitors (satellite cells) as compared to young muscle (Day et al., 2010). The decreased number of myogenic progenitors may be due to the activation of quiescent satellite cells, which in response to myofiber damage, proliferate and migrate to regenerate the injured myofibers throughout life (Blau et al., 2015). Although the quiescent satellite cell pool is maintained via asymmetric division, the number of satellite cells is gradually depleted with aging (Kuang et al., 2007; Sousa-Victor et al., 2014). Nevertheless, ~ 70% of the satellite cells from old mice are not senescent (Cosgrove et al., 2014) and are still capable of proliferation and differentiation (Shefer et al., 2006).

Almost 90% of the outgrowth cells on the matrigel coated dish were myogenic progenitors (α7-integrin+, SCA-1/CD31/CD45−, MyoD+, Desmin+ cells) (Fig. 2). However, collagen coating yielded minimal outgrowth of cells from tissues, with most of the cells exhibiting fibroblastic phenotype (Fig. S4). This preferential adhesion of fibroblasts to collagen was used to increase the purity of outgrowth myoblasts to > 98% (Fig. 3A–C), eliminating the need for downstream purification by FACS sorting.

The main component of matrigel is laminin, which can mediate the adhesion of myogenic cells through the muscle specific integrin, α7β1 (Yao et al., 1996; Riederer et al., 2015). Laminin is known to be essential for migration and proliferation of murine myoblasts (Fig. S2B) (Ocalan et al., 1988). In addition, recent studies showed that laminin promotes proliferation of human myoblasts (Chowdhury et al., 2015; Soriano-Arroquia et al., 2017) and maintains their myogenic phenotype over long-term culture (Penton et al., 2016), suggesting that this isolation protocol may also be applicable to human skeletal muscle myoblasts. The high serum medium supplemented with CEE and bFGF facilitated the migration and expansion of myoblasts on matrigel. Although not present in the proliferation media of previous reports (Wang et al., 2014), we observed that bFGF promoted proliferation and inhibited spontaneous differentiation of myoblasts (Fig. S2A).

The proliferating myoblasts could be expanded for > 10 passages with a doubling time of 24 ± 1 h and minimal spontaneous differentiation (Fig. 3B&D). Furthermore, the mRNA expression profile of myogenic regulatory factors suggests that the cells in culture were mostly proliferating myoblasts as evidenced by higher levels of early myogenic regulatory factors *Myf5* and *MyoD* and lower levels of late differentiation markers, *MyoG* and *MRF4* (Fig. 3E) (Yablonka-Reuveni, 2011; Zanou and Gailly, 2013; Shahini et al., 2018). Upon reaching confluence, the myoblasts exhibited pronounced myogenic differentiation capacity as evidenced by formation of multinucleated myotubes capable of spontaneous contraction. The myotubes were positive for MYHC and Actinin, and formed sarcomeres (Fig. 4).

Overall, we describe a robust and high-yield protocol to isolate myoblasts from young and old murine skeletal muscle. Our protocol eliminates the steps of cell straining and cell sorting, and maximizes the total number of myoblasts isolated from the tissues by enabling outgrowth of myogenic progenitors from their native niche. We show that enzymatic digestion, although not necessary, accelerates the outgrowth of myogenic progenitors. However, matrigel in combination with high serum and bFGF are necessary for myoblast outgrowth and expansion. The purity of myoblasts is further enhanced by removal of fibroblasts upon pre-plating on collagen as well as the proliferation advantage afforded to myoblasts by the proliferation medium.

Supplementary data and materials and methods to this article can be found online at https://doi.org/10.1016/j.scr.2018.05.017.

## Supplementary Material

1

2

3

## Abbreviations

FACS: fluorescence activate cell sorting
MACS: magnetic-activate cell sorting
EHS: Engelbreth-Holm-Swarm
TGF-β: transforming growth factor beta
EGF: epidermal growth factor
IGF1: insulin-like growth factor
PDGF: platelet derived growth factor
bFGF: basic fibroblast growth factor
CEE: Chicken Embryo Extract
PBS: phosphate buffer saline
PM: proliferation medium
DM: Differentiation Medium
FBS: fetal bovine serum
HS: Horse Serum

## References

[R1] Allbrook DB, Han MF, Hellmuth AE (1971). Population of muscle satellite cells in relation to age and mitotic activity. Pathology.

[R2] Bareja A, Billin AN (2013). Satellite cell therapy – from mice to men. Skelet. Muscle.

[R3] Blau HM, Cosgrove BD, Ho ATV (2015). The central role of muscle stem cells in regenerative failure with aging. Nat. Med.

[R4] Calve S, Odelberg SJ, Simon H-G (2010). A transitional extracellular matrix instructs cell behavior during muscle regeneration. Dev. Biol.

[R5] Chowdhury SR, binti Ismail A, Chee SC, bin Laupa MS, binti Jaffri F, Saberi SE, Idrus RB (2015). One-step purification of human skeletal muscle myoblasts and subsequent expansion using laminin-coated surface. Tissue Eng. Part C Meth.

[R6] Cosgrove BD, Gilbert PM, Porpiglia E, Mourkioti F, Lee SP, Corbel SY, Llewellyn ME, Delp SL, Blau HM (2014). Rejuvenation of the muscle stem cell population restores strength to injured aged muscles. Nat. Med.

[R7] Danoviz ME, Yablonka-Reuveni Z (2012). Skeletal muscle satellite cells: background and methods for isolation and analysis in a primary culture system. Meth. Mol. Biol. (Clifton, N.J.).

[R8] Day K, Shefer G, Shearer A, Yablonka-Reuveni Z (2010). The depletion of skeletal muscle satellite cells with age is concomitant with reduced capacity of single progenitors to produce reserve progeny. Dev. Biol.

[R9] Fischedick G, Wu G, Adachi K, Araúzo-Bravo MJ, Greber B, Radstaak M, Köhler G, Tapia N, Iacone R, Anastassiadis K, Schöler HR, Zaehres H (2014). Nanog induces hyperplasia without initiating tumors. Stem Cell Res.

[R10] Gharaibeh B, Lu A, Tebbets J, Zheng B, Feduska J, Crisan M, Peault B, Cummins J, Huard J (2008). Isolation of a slowly adhering cell fraction containing stem cells from murine skeletal muscle by the preplate technique. Nat. Protoc.

[R11] Goetsch KP, Snyman C, Myburgh KH, Niesler CU (2015). Simultaneous isolation of enriched myoblasts and fibroblasts for migration analysis within a novel co-culture assay. BioTechniques.

[R12] Grefte S, Vullinghs S, Kuijpers-Jagtman AM, Torensma R, Hoff JWVD (2012). Matrigel, but not collagen I, maintains the differentiation capacity of muscle derived cells in vitro. Biomed. Mater.

[R13] Hindi L, McMillan JD, Afroze D, Hindi SM, Kumar A (2017). Isolation, culturing, and differentiation of primary myoblasts from skeletal muscle of adult mice. Bio. Protoc.

[R14] Hughes CS, Postovit LM, Lajoie GA (2010). Matrigel: a complex protein mixture required for optimal growth of cell culture. Proteomics.

[R15] Icronimakis NB, Gayathri, Chamberlain, Jeffrey S, Reyes, Morayma (2007). 139. Isolation, characterization, and myogenesis of satellite cells derived from skeletal muscle. Mol. Ther.

[R16] Kuang S, Kuroda K, Le Grand F, Rudnicki MA (2007). Asymmetric self-renewal and commitment of satellite stem cells in muscle. Cell.

[R17] Motohashi N, Asakura Y, Asakura A (2014). Isolation, culture, and transplantation of muscle satellite cells. J. Visual. Exp.

[R18] Ocalan M, Goodman SL, Kuhl U, Hauschka SD, von der Mark K (1988). Laminin alters cell shape and stimulates motility and proliferation of murine skeletal myoblasts. Dev. Biol.

[R19] Osorio FG, Navarro CL, Cadinanos J, Lopez-Mejia IC, Quiros PM, Bartoli C, Rivera J, Tazi J, Guzman G, Varela I, Depetris D, de Carlos F, Cobo J, Andres V, De Sandre-Giovannoli A, Freije JM, Levy N, Lopez-Otin C (2011). Splicing-directed therapy in a new mouse model of human accelerated aging. Sci. Transl. Med.

[R20] Pasut A, Jones AE, Rudnicki MA (2013). Isolation and Culture of Individual Myofibers and their Satellite Cells from Adult Skeletal Muscle.

[R21] Penton CM, Badarinarayana V, Prisco J, Powers E, Pincus M, Allen RE, August PR (2016). Laminin 521 maintains differentiation potential of mouse and human satellite cell-derived myoblasts during long-term culture expansion. Skelet. Muscle.

[R22] Piazzolla D, Palla AR, Pantoja C, Canamero M, de Castro IP, Ortega S, Gomez-Lopez G, Dominguez O, Megias D, Roncador G, Luque-Garcia JL, Fernandez-Tresguerres B, Fernandez AF, Fraga MF, Rodriguez-Justo M, Manzanares M, Sanchez-Carbayo M, Garcia-Pedrero JM, Rodrigo JP, Malumbres M, Serrano M (2014). Lineage-restricted function of the pluripotency factor NANOG in stratified epithelia. Nat. Commun.

[R23] Rando TA, Blau HM (1994). Primary mouse myoblast purification, characterization, and transplantation for cell-mediated gene therapy. J. Cell Biol.

[R24] Riederer I, Bonomo AC, Mouly V, Savino W (2015). Laminin therapy for the promotion of muscle regeneration. FEBS Lett.

[R25] Sarrazin S, Lamanna WC, Esko JD (2011). Heparan sulfate proteoglycans. Cold Spring Harb. Perspect. Biol.

[R26] Shahini A, Choudhury D, Asmani M, Zhao R, Lei P, Andreadis ST (2018). NANOG restores the impaired myogenic differentiation potential of skeletal myoblasts after multiple population doublings. Stem Cell Res.

[R27] Shefer G, Yablonka-Reuveni Z (2005). Isolation and culture of skeletal muscle myofibers as a means to analyze satellite cells. Meth. Mol. Biol. (Clifton, N.J.).

[R28] Shefer G, Van de Mark DP, Richardson JB, Yablonka-Reuveni Z (2006). Satellite-cell pool size does matter: defining the myogenic potency of aging skeletal muscle. Dev. Biol.

[R29] Soriano-Arroquia A, Clegg PD, Molloy AP, Goljanek-Whysall K (2017). Preparation and Culture of Myogenic Precursor Cells/Primary Myoblasts from Skeletal Muscle of Adult and Aged Humans.

[R30] Sousa-Victor P, Gutarra S, Garcia-Prat L, Rodriguez-Ubreva J, Ortet L, Ruiz-Bonilla V, Jardi M, Ballestar E, Gonzalez S, Serrano AL, Perdiguero E, Munoz-Canoves P (2014). Geriatric muscle stem cells switch reversible quiescence into senescence. Nature.

[R31] Wang Z, Cheung D, Zhou Y, Han C, Fennelly C, Criswell T, Soker S (2014). An in vitro culture system that supports robust expansion and maintenance of in vivo engraftment capabilities for myogenic progenitor cells from adult mice. BioRes. Open Access.

[R32] Yablonka-Reuveni Z (2011). The skeletal muscle satellite cell: still young and fascinating at 50. J. Histochem. Cytochem.

[R33] Yao C-C, Ziober BL, Squillace RM, Kramer RH (1996). α7 integrin mediates cell adhesion and migration on specific laminin isoforms. J. Biol. Chem.

[R34] Yi L, Rossi F (2011). Purification of progenitors from skeletal muscle. J. Visual. Exp.

[R35] Yin H, Price F, Rudnicki MA (2013). Satellite cells and the muscle stem cell niche. Physiol. Rev.

[R36] Zanou N, Gailly P (2013). Skeletal muscle hypertrophy and regeneration: interplay between the myogenic regulatory factors (MRFs) and insulin-like growth factors (IGFs) pathways. Cell. Mol. Life Sci.

