# Supplementary material for: Efficient and high yield isolation of myoblasts from skeletal muscle
Source: Stem Cell Res. Author manuscript; Available in PMC 2018 Aug 14. (PMC6090567; doi:10.1016/j.scr.2018.05.017)
Supplement: 3 [file NIHMS1500572-supplement-3.docx]

**Supplementary Materials and Methods**

**Flow Cytometry**

To perform flow cytometry, hindlimb muscles were minced and digested using collagenase type II (500U/ml), collagenase D (1.5U/ml), and dispase II (2.5U/ml) in the presence of 2.5mM CaCl_2_ and incubation for 60 min in 37°C water bath with frequent agitations every 5 min. Subsequently, 40ml of PBS was added and mixed with the muscle slurry. The suspension was passed through a 70µm cell strainer to filter out the remaining large pieces of skeletal muscle and centrifuged at 300xg for 5 min to pellet the mononuclear cells. The pellet was resuspended in Flow Cytometry Staining Buffer (FCSB, R&D SYSTEMS, Minneapolis, Minnesota) and the following antibodies were added as follows: Sca-1/FITC at 0.5µg/test, CD45/FITC 0.5µg/test, CD31/FITC at 1 µg/test, and α7-integrin/APC at 10µl/test (all antibodies were purchased from ThermoFisher Scientific, Waltham, MA). After incubation at 4ºC for 1hr, the sample were washed twice with FCSB and centrifuged at 300xg. The pellet was resuspended in 200µl FCSB and flow cytometry was performed using BD Fortessa X20, four-laser, 16-color analyzer. The results were analyzed using FCS Express 6 Flow.

**Immunostaining**

Cells were fixed in 4% paraformaldehyde for 10 min at room temperature (RT) before permeabilized with 0.1% (v/v) triton X-100/PBS for 10 min at RT and blocked with blocking buffer (5% (v/v) goat serum in 0.01% (w/v) triton X-100/PBS) at 25°C for 1hr. Then, samples were probed for alpha smooth muscle actin (αSMA, 1:100 dilution in blocking buffer overnight, Sigma-Aldrich, St. Louis, MO); MyoD (1:200 dilution in blocking buffer overnight, BD Biosciences, San Jose, CA); Desmin (1:200 in blocking buffer overnight; Cell Signaling); Myosin Heavy Chain (1:1,000 dilution in blocking buffer overnight, Millipore, Billerica, MA); Actinin (1:200 dilution in blocking buffer overnight, Abcam). Subsequently, the cells were stained with Alexa Fluor 594 or 488 conjugated goat anti-mouse or goat anti-rabbit antibodies (1:200 dilution in blocking buffer for 1hr, ThermoFisher Scientific) and counter-stained with Hoechst 33342 nuclear dye for 5 min (1:400 dilution in PBS, Thermo Fisher Scientific). Images of cells were acquired using Zeiss Axio Observer Z1 (LSM 510; Zeiss, Oberkochen, Germany) equipped with digital camera (ORCA-ER C4742-80; Hamamatsu, Bridgewater, NJ). Image quantification was performed by counting the number of positive cells in 7 different images and the number of cells and p-values are stated in the text and figure legends.

**Staining with Hematoxylin and Eosin:**

The fixed cells were stained with Hematoxylin for 1min, washed with water for 5min, stained with Eosin for 3 min, then washed with water for 3 min, and imaged using EVOS *fl* inverted digital microscope.

**RNA Isolation, cDNA Synthesis, and Quantitative Real Time PCR (qRT-PCR):**

RNA of the cells in culture were isolated using RNeasy Mini Kit (QIAGEN, Valencia, CA) according to the manufacturer’s protocol. cDNA was synthesized using High-Capacity cDNA Reverse Transcription Kit (Thermo Fisher Scientific, Waltham, MA). Power SYBR Green PCR Master Mix (Thermo Fisher Scientific) was used to perform qRT-PCR and the mRNA expression was calculated based on the ΔC_T_ method and normalized to the house keeping gene *Polr2b*. The values are mean ± std of triplicates in each experiment. The sequences of primers were obtained from PrimerBank and are listed below (5’🡪3’):

*Myf5*;Forward: GCCTTCGGAGCACACAAAG, Reverse: TGACCTTCTTCAGGCGTCTAC

*MyoD*; Forward: ATGATGACCCGTGTTTCGACT, Reverse: CACCGCAGTAGGGAAGTGT

*MyoG*; Forward: GAGACATCCCCCTATTTCTACCA, Reverse: GCTCAGTCCGCTCATAGCC

*MRF4*; Forward: CTGAAGCGTCGGACTGTGG, Reverse: ATCCGCACCCTCAAGAATTTC

*Polr2b*; Forward:GGTCAGAAGGGAACTTGTGGTAT, Reverse:GCATCATTAAATGGAGTAGCGTC

**Video Recording:**

Myotube contractions were captured using Zeiss Axio Observer Z1 at 10 frame per second. The video of protocol was recorded by Canon VIXIA HF R80 camera and the videos were collated using iMovie software version 10.1.8.
